# Prevalence and Clinical and Immunoviralogical Profile of Human Immunodeficiency Virus-Hepatitis B Coinfection among Children in an Antiretroviral Therapy Programme in Benue State, Nigeria

**DOI:** 10.1155/2013/932697

**Published:** 2013-04-03

**Authors:** Emmanuel Ademola Anigilaje, Ayodotun Olutola

**Affiliations:** ^1^Department of Paediatrics, Federal Medical Centre, PMB 12245, Makurdi, Benue State, Nigeria; ^2^Center for Clinical Care and Clinical Research, 29 Mambilla Street, Off Aso Drive, Maitama, Abuja, Nigeria

## Abstract

*Background*. Nigeria has the world largest burden of paediatric HIV and is also highly endemic for Hepatitis B virus (HBV). However, relatively little is known regarding the prevalence of HBV-HIV coinfections among Nigerian children. *Methods*. A retrospective study among treatment naive HIV-infected children attending the pediatric clinic of the APIN Plus/Harvard PEPFAR program of the Federal Medical Centre, Makurdi, between June 2008 and June 2012. *Results*. The mean age of the 395 subjects studied was 7.53 ± 4.23 years. Thirty-one subjects (7.8%) were positive for HBV. No subject was HIV-HBV-HCV triply infected. Significantly higher HIV-HBC coinfections were found, in older subjects (11–15 years), subjects that did not receive nor complete Hepatitis B vaccinations, and subjects that had a severe immunosuppression of < 15% with respective *P* values of 0.00, 0.01, and 0.00. HIV-HBV co-infection did not significantly impact on other baseline characteristics including, gender, WHO clinical stage, median absolute CD4 count, mean viral load, median ALT, and hepatotoxicity. *Conclusion*. A high seroprevalence of HBV among this cohort of HIV-infected children contributes to the calls for pre-ART screening for HBV and the necessary paradigm shift in the ART nucleoside backbone to include agent(s) more dually effective against HIV and HBV.

## 1. Introduction

With an estimation of 250,000 children infected with HIV, Nigeria accounts for more than 10% of the global paediatric HIV burden [1]. More than 90% of these infections were vertically acquired from mother to child. Nigerian coverage of Prevention of Mother to Child Transmission (PMTCT) strategies has meagerly increased from 5.3% in 2007 to 11% in 2010 [2]. This implies that many new paediatric infections continue to occur, with Nigeria accounting for 30% of the global PMTCT gaps [1]. Regional differences in the HIV burden exist in Nigeria. Benue State, located in the North Central region, had the highest estimated HIV prevalence of 12.7% in 2010 [2–4]. Several other pathogens including Hepatitis B virus are acquired vertically with the attendant risks of coinfections and the complex interactions.

Nigeria is also known to be highly endemic for Hepatitis B viral (HBV) infection [5]. There is a relative paucity of data on HIV/HBV coinfection. A few studies have reported rates ranging from prevalence of 7.7%, to 19% of HIV-HBV coinfections in Nigerian children [6–8]. Precariously, the prevalence of pediatric HBV-HIV coinfections may be higher as most HBV infections occur within the first 5 years of life in children in Africa [9]. In addition, Mbaawuaga et al. [10] had earlier demonstrated a high sero-prevalence of 11% of HBV infection with infectivity rates of 3.3% among pregnant women in Makurdi, Benue State. Complex interactions between HIV and HBV have been well documented in adult population [11–18]. Higher HBV DNA levels have been found in those coinfected with HIV and HBe antibody seroconversion occurring less frequently in HIV-coinfected individuals, thereby delaying transition to the inactive carrier state with its attendant higher risk of advanced liver disease [11–14]. The impact of HBV on HIV disease is less clear. Whilst one study showed an increased rate of HIV progression to AIDS [15], others investigators did not show any change in the progression of HIV disease or survival [16]. However, coinfection with HBV has been associated with increased hepatotoxicity to highly active antiretroviral therapy (HAART) [17, 18]. With easy access to and success of HAART in reducing mortality from AIDS, longevity means that coinfected children are more exposed to the aforementioned complex interactions. The need to document the burden of HIV-HBV coinfections in Nigerian children like any others can no longer be overemphasized. This study therefore determined the prevalence proportion, clinical, demographic, and immunovirological characteristics of HIV-HBV coinfections among children receiving care and treatment for HIV/AIDS in a tertiary health centre in Makurdi, Benue State, Nigeria.

## 2. Methods

The study was carried out among HIV-infected children receiving care and treatment at the Paediatric ART (Antiretroviral therapy) Clinic of the Riverside Specialist Clinics of the Federal Medical Centre (FMC), Makurdi. FMC, Makurdi, is the only tertiary health hospital providing care and treatment for paediatric HIV in Benue State and, therefore, is a referral centre for primary and secondary health facilities in Benue State and the surrounding states of Taraba, Nasarawa, and Kogi. The facility is supported by the AIDS Prevention Initiative in Nigeria (APIN)/Harvard PEPFAR (The USA President's Emergency Plan for AIDS Relief) program providing ART to HIV-infected children according to the Nigerian Guidelines on Paediatric HIV/AIDS Treatment and Care. Ethical approval for the study was obtained from the Hospital Research and Ethics Committee. HIV-infected children are recruited into care and treatment upon consent of parents/caregivers and assent of the child if older than age of 7 years. Inclusion criteria included subjects who were screened and got results for Hepatitis B surface antigen and HCV antibody at the time of the HIV diagnosis and before the commencement of ART over the study period, between June 2008 and June 2012. A study proforma was designed to extract the following information from the subjects' data bank at recruitment: age, sex, immunization history, mode of transmission of HIV, WHO clinical staging, CD4+ counts, CD4%, HIV viral load, and alanine transaminase (ALT) level. All subjects ≥18 months had an initial double rapid HIV antibody tests using Determine HIV 1/2 (by Abbott Japan Co., Ltd. Minato-Ku, Tokyo, Japan) first and then HIV 1/2STAT-PAK (by Chembio Diagnostics Systems, Inc., Medford, NY 11763, USA) in serial algorithm. HIV infection was confirmed in subjects who had reactive rapid test by using a Western Blot test. Two HIV DNA PCR positivity tests for those <18 months confirmed HIV infection in this age group. Detection of HBsAg and HCV was done using the third generation ELISA technique for both HBsAg (EIAgen HBsAg Kit) and HCV antibody (EIAgen HCV Ab Kit). The tests were done according to the instructions of the manufacturer (Diaspot, USA). Catalytic activity of ALT was determined in serum using a Cobas Mira chemistry analyzer (GMI, MI, USA) after it was calibrated. All tests were done in the APIN/PEPFAR laboratory of the hospital.

### 2.1. Definitions

HBsAg status is defined as either HBsAg positive/HIV-HBV co-infected or HBsAg negative. Hepatotoxicity was defined as ALT 1.25-fold over the upper limit of normal (ULN is 37 iu/l) [19]. Levels of immunological suppression were defined as severe when the CD4% <15, moderate when the CD4% is between 15 and 25, and mild when CD4% >25. Only one or two doses of Hepatitis B vaccine were considered to be incomplete, and three doses were considered to be complete.

### 2.2. Data Analysis

Data were analyzed using SPSS version 19. The overall seroprevalence of HBsAg was expressed in percentages and by age and gender. Chi-Square (*χ* ^2^) was used to determine the association between HBsAg status and age groups, gender, HBV immunization status, WHO clinical stages, and hepatotoxicity. Fisher's exact test was employed to test the association between HBsAg status and routes of HIV acquisitions and ranges of CD4% immunosuppression. Plotted histogram and normal probability curves were used to test for normality of CD4 counts, viral load, and ALT, and only viral load was found to be normally distributed. Medians of CD4 counts and ALT levels of recruitment between HIV-monoinfected and HIV-HBV-coinfected groups were compared using the Mann-Whitney u test. Also, the means of age and the means of Log10 of viral load were compared using unpaired *t*-test between HIV-monoinfected and HIV-HBV-coinfected groups. *P* < 0.05 was taken as significant.

## 3. Results

### 3.1. Clinical and Demographic Characteristics of Subjects (Table 1)

A total of 936 children were seen within the study period (from June 2008 to June 2012), but only 395 subjects were screened and received results for viral Hepatitis B and C. The remaining 541 were not screened for reasons bordering on failure to request for screening for the viral hepatitis and occasional instances when the testing kits went out of stock. Among the 395 subjects screened, 31 subjects (7.8%) were positive for HBV. Nine subjects (2.3%) were positive for Hepatitis C. No subject was positive for both HBV and HCV. The mean age for the 395 subjects was 7.53 ± 4.23 years. The age range was from 8 months to 15 years. There were 205 males and 190 females with an M : F ratio of 1 : 0.9. The majority, 159, was less than 5 years, while others were fairly distributed within the age group of 6–10 years (122) and 11–15 years (114). 

### 3.2. Clinical, Demographic, and Laboratory Characteristics of HIV-HBV Coinfection Status Groups (Table 2)

In order to remove the possible confounding effect of HCV infection, only 386 subjects (excluding 9 who tested positive for HCV) were tested for the effect of HIV-HBV co-infection on WHO clinical stage, CD4 counts and CD4%, viral load, and ALT. Out of these 386 subjects, 108 (27.9%) data were missing for CD4 count/CD4%, 118 (30.6%) data were missing for viral load, and only 8 data (2.1%) were missing for ALT. Again, reasons for missing data included occasional instances when testing kits went out of stock, insufficient and spilled blood samples, and equipment breakdown especially during the early period of the program.

### 3.3. HIV-HBV Coinfections by Age and Gender

The mean ages of 9.74 ± 4.08 and 7.34 ± 4.19 years were compared between dually infected and HIV-monoinfected subjects, respectively, and were significant, (*P* value 0.00). The majority, (17, 14.9%) of HIV-HBV viral co-infections were in the 11–15 year group and were significant (*P* value 0.00). Although more female subjects (19, 10%) were dually infected with HIV and HBV than male subjects (12, 5.9%), this was not statistically significant (*P* value 0.18).

### 3.4. HIV-HBV Coinfections and HBV Immunizations

Hepatitis B viral co-infection was found among subjects that did not receive or complete Hepatitis B vaccinations (20, 6.1%) compared to subjects that had (11, 16.7%) the three doses of Hepatitis B vaccinations, and this was found to be significant (*P* value 0.01). 

### 3.5. HIV-HBV Coinfections and Route of HIV Acquisitions

The majority of the subjects (27, 7.9%) that were co-infected with Hepatitis B viral infection also acquired the HIV infection via Mother to Child Transmission; this was not statistically significant. Only one subject (14.3%) who acquired HIV infection via blood transfusion was also coinfected with HBV. The route of acquisition of HIV for three subjects with HIV-HBV viral coinfection could not be ascertained: *P* value of 0.55.

### 3.6. HIV-HBV Coinfections and WHO Clinical Staging

Although the majority of HIV-HBV-co-infected subjects (21, 7.7%) were recruited in WHO clinical stages 1 and 2, the finding was not statistically significant (*P* value 0.91).

### 3.7. HIV-HBV Coinfections and Baseline Laboratory Findings

 A lower median absolute CD4 count of 334 cells/mL (IQR from 182.50 to 830.50) was found among HIV-HBV co-infected subjects compared to a median of 560 cells/mL (IQR from 275.00 to 916.80) among HIV-monoinfected subjects (*P* value 0.11). Furthermore, 15.8% (12) of HIV-HBV-coinfected subjects had a severe immunosuppression of <15% and was significant (*P* value 0.00). 

The mean viral load was comparable among both groups with 4.26 ± 1.29 among the HIV-HBV co-infected subjects and 4.51 ± 1.17 among the HIV-monoinfected subjects but was not significant (*P* value 0.33). 

A higher median of ALT value of 30.20 (IQR from 18.50 to 50.70) among the HIV-HBV-co-infected subjects was compared to a median of 24.70 (IQR from 16.65 to 35.32) among the HIV-monoinfected group and was not statistically significant (*P* value 0.08). Normal ALT values were found more among HIV-monoinfected subjects (270, 93.1%) compared to the HIV-HBV-coinfected subjects (20, 6.9%), and hepatotoxicity was lower among HIV-HBV-coinfected subjects (11, 12.5%) compared to 77 (22.2%) among HIV-monoinfected subjects; this was also not found to be significant (*P* value 0.43).

## 4. Discussion

The prevalence of 7.8% for Hepatitis B surface antigen in this study is similar to 7.7% described by Sadoh et al. [6] in Benin, Nigeria, but higher than the respective sero-prevalence of 1.2%, 2.6%, 4%, and 4.9% reported by Telatela et al. [20] in Tanzania, Toussi et al. [21] in New York, USA, Chakraborty et al. [22] in Kenya, and Zhou et al. [23] in China, in similar cohorts of HIV-infected children. Furthermore, the prevalence of 7.8% in this study is lower than the respective 8.4%, 19%, and 12.1% reported by Rawizza et al. [7] in Nigeria, Ashir et al. [8] in Maiduguri, Nigeria, and Rouet et al. [9] in Ivory Coast also in cohorts of pediatric HIV-infected children. Differences in Hepatitis B surface antigenemia among different populations of children have been noted previously [6]. Thus, the prevalence of 7.8% for HBsAg in this study is also higher than 1.8% reported by Jafri et al. [24] in children in Karachi, Pakistan, and 4.1% of Ugwuja and Ugwu [25] in Abakaliki, South Eastern Nigeria. Ndako et al. [26] had earlier found a higher prevalence of 9.7% of Hepatitis B surface antigenemia among children in Sabon Pegi, Karu, Nigeria. Furthermore, Angyo and Yakubu [27] also reported a much higher prevalence of 19.6% and 22.7% among respective children with haemoglobin AA and haemoglobin SS in Jos, Nigeria. Above all, Bukbuk et al. [28] reported a prevalence of 44.7% among pupils in primary school in rural Borno, Nigeria.

Whereas the shared modes of transmission of HBV and HIV would explain the high prevalence of HBsAg among HIV-infected children in our study and those of others [6–9, 20–23], the differences in subjects' selection as well as diverse socioeconomic and demographic risk factors that favored horizontal transmissions of HBV in the different settings of the non-HIV-infected children may explain the different seroprevalence of HBV in other studies [24–28]. For example, unsafe injection from unqualified medical personnel using HBV contaminated needle and syringe, transfusion of blood and blood products, and sociocultural practices such as tribal marks, circumcision, and scarification were important routes of HBV transmission in the study by Ugwuja and Ugwu [25]. Jafri et al. [24] in Pakistan also identified inappropriate injections using new or reused needles and syringes and the use of multiple-dose vials as horizontal risk factors for HBV contraction. However, Angyo and Yakubu [27] did not find a significant association between HB surface antigenemia and the potential risk factors including blood transfusion, parenteral injections, hospitalization, traditional uvulectomy, circumcision, ear piercing, traditional scarifications, and contacts with case of known Hepatitis in cohorts of children with sickle cell anaemia and those with haemoglobin AA. 

A significant number of our subjects (17, 14.9%) that were seropositive for HBV were found to be adolescents between the ages of 11–15 years. A similar trend was noticed by Zhou et al. [23] in China whereby children older than 11 years were significantly more infected with HBV. Toussi et al. [21] in New York, USA, also found that children who are HIV-HBV-coinfected had a higher median age of 17 years compared to HIV-monoinfected children with a median age of 11.4 years. Also in Tanzania [20], the only 2 children that were seropositive for HBsAg was in the age group of 6–10 years, although this finding was not statistically significant. In contrast, there was no age group risk of sero-positivity for HBs Ag in the study by Sadoh et al. [6]. In Nigeria, Hepatitis B vaccine was first introduced as part of the National Program on Immunization in 2004, and this may partly explain the reason for a higher HBs Ag sero-positivity among subjects within 11–15 years group. This assumption could have been buttressed by the fact that HBV infection was also found to be significantly more among our subjects who had none or did not complete HBV immunization. However, since the introduction of Hepatitis B vaccine in 2004, its coverage has increased steadily from 18% in 2005 to 66% in 2010 with an unfortunate decline to 50% in 2011 [29]. Another potential route of transmission, especially through sexual exposure, may be difficult to ascertain among adolescents. Although the majority of our subjects (27, 7.9%) also appears to have acquired HIV via MTCT, this finding was not significant, and only 6 subjects (3.8%), co-infected with HBV, were 5 years and below. Mindful of the shared mode of transmission of HIV and HBV, vertical transmission of HBV cannot be substantiated in our study as more subjects less than 5 years of age would have been expected to be HIV-HBV co-infected. Although Mbaawuaga et al. [10] had demonstrated a high sero-prevalence of 11% of HBV infection among pregnant women in Makurdi, the low infectivity rate of 3.3%, a major determinant of perinatal transmission found in their study, may also have explained this low prevalence of HBV among the underfives in our study.

More female subjects (19, 10%) were dually infected with HIV and HBV than male subjects (12, 5.9%), but this was not statistically significant. The import of this finding may not be readily discernible.

Our data indicated that the majority of, albeit insignificant, HIV-HBV-coinfected subjects were recruited early in WHO Clinical stages 1 and 2 disease but presented with a lower median CD4 count. A significant majority of the HIV-HBV co-infected children in the present study, also presented at a much reduced CD4 of ≤ 15%. Although, individuals with WHO clinical stages 1 and 2 have been well known to have substantially reduced CD4% and thus may explain the discordance between the CD4% and the WHO clinical stages as also found in the present study. In Rawizza et al. [7] study, CD4 cell count and WHO clinical stage were not significantly different between HIV-HBV-coinfected and HIV-monoinfected children.

The effect of HIV-HBV co-infections on HIV replication in our study is not clearly defined as the mean viral load among the HIV-HBV-coinfected subjects was insignificantly lower than that among the HIV-monoinfected subjects. Toussi et al. [21] in New York, however, showed undoubtedly that HIV-HBV-coinfected children tended to be more symptomatic (CDC category C) and had a lower CD4% and a higher HIV RNA levels than their monoinfected counterparts.

Although other accompanying opportunistic infections may also have explained the reduced CD4 count among our HIV-HBV-coinfected patients, this confounding factor was outside the scope of present study.

In this study, a higher median and hepatotoxic ALT values among the HIV-HBV-coinfected subjects compared to the HIV-monoinfected group were not statistically significant. Similar data were obtained by Rawizza et al. [7] who also did not find significant differences in elevated ALT between the two HBV status groups. In China, Zhou et al. [23] also reported that although mildly elevated ALT was higher in HIV-HBV-coinfected group compared to HIV-monoinfected counterpart, the difference was not significant. In Tanzania [20] study, elevated ALT value was associated with HIV-HBV co-infections in the univariate analysis but not in multivariate analysis. Although longitudinal study of the impact of HBV on ALT activities among our HIV-infected children in the long term is desirable, HBV has been reported earlier [30] to be a cause of elevated liver enzymes as also found in this study. Liver biopsy, the gold standard for assessing disease severity in HIV-HBV coinfection, is however outside the scope of this study. 

In most developing countries including Nigeria and in our center, the first line HAART for our patients included a regimen containing 3TC/AZT backbone. Although, the efficacy of 3TC against HBV has been proven by Dore et al. [31] with the CAESAR study, 3TC based regimen had also been associated with a rapid development of resistance to HBV [32]. These findings have influenced the evolution of new treatment algorithms, wherein TDF and 3TC/FTC are included as the nucleoside backbone, irrespective of prior exposure to lamivudine [33]. This new nucleoside back is associated with a good HBV DNA suppression and normalization of the alanine transaminase level [33]. Fortunately, TNF is now approved for use in special circumstances (possibly HIV-HBV coinfection) among children from the age of 2 years [34]. 

 A paradigm shift to include this new nucleoside backbone as part of first line HAART may become inevitable as this study and those of others [6–8] had shown consistently a high prevalence of HIV-HBV co-infection among Nigerian children.

## 5. Conclusion

The present study had revealed a high prevalence of 7.8% of HBsAg among our HIV-infected children and underscored the predominance of HBV infection among young adolescent subjects aged 11–15 years and the import of HBV immunization as significantly more subjects with none or incomplete HBV vaccination were infected. It also revealed that HBs antigenemia occurred significantly more among subjects with severe immunosuppression. 

It is therefore recommended that all pediatric HIV-infected patients should be screened for HBV on recruitment into care and treatment. Also, efforts aimed at increasing the coverage and completeness of HB vaccination via the strengthening of the Nigerian Expanded Program on Immunization can also not be overemphasized. Moreover, antenatal screening of HBV among pregnant women should be intensified, and the neonate of those found positive should be offered both passive and active immunization. Furthermore, longitudinal study on the impact of HIV-HBV co-infection on immune recovery, HIV and HBV viral replication, and hepatotoxicity over time is desirable and also recommended. 

## Figures and Tables

**Table 1:** Clinical and demographic characteristics of subjects.

Characteristics	Number	Percentage
Mean age ± standard deviation	7.53 ± 4.23	
Gender		
Male	205	51.9
Female	190	48.1

Total	395	100.0

Age groups in years		
<5	159	40.3
6–10	122	30.8
11–15	114	28.9

Total	395	100.0

Hepatitis BsAg status groups		
Positive (HIV-HBV dually infected)	31	7.8
Negative	364	92.2

Total	395	100.0

Hepatitis C antibody status groups		
Positive (HIV-HCV dually infected)	9	2.3
Negative	386	97.7

Total	395	100

**Table 2:** Clinical, demographic, and laboratory characteristics of HIV-HBV coinfection status groups.

Characteristics	Hepatitis BsAg positive (HIV-HBV dually infected)	Hepatitis BsAg negative	Missing data	*P* value
Mean age in years ± SD	9.74 ± 4.08	7.34 ± 4.19		0.00
Age group in years				
≤5	6	153		0.00
6–10	8	114		
11–15	17	97		

Total	31	364		

Gender				
Male	12	193		0.18
Female	19	171		

Total	31	364		

HBV immunization				
None/incomplete	20	309		0.01
Complete	11	55		

Total	31	364		

Route of HIV acquisitions				
MTCT	27	339		0.55
Blood transfusion	1	6		
Others (sexual)	0	2		
Unknown	3	17		

Total	31	364		

WHO staging*				
1 and 2	21	250		0.91
3 and 4	10	105		

Total	31	355		

Absolute CD4 count in cells/mm, median (IQR)*	334.00 (182.50–830.50)	560.00 (275.00–916.80)	108	0.11
CD4%*				
<15	12	64		0.00
15–25	2	50		
>25	6	144		

Total	20	258		

Log_10_ viral load, Mean ± SD*	4.26 ± 1.29	4.51 ± 1.17	118	0.33
ALT, Median (IQR)*	30.20 (18.50–50.70)	24.70 (16.65–35.32)	8	0.08
Hepatotoxicity*				
Normal	20	270		0.43
Elevated	11	77		

Total	31	347		

SD: standard deviation.

IQR: interquartile range.

*Data of 9 subjects (HIV-HCV coinfected) were excluded.
